# Inhibition of mitochondrial cyclophilin D, a downstream target of glycogen synthase kinase 3α, improves sperm motility

**DOI:** 10.1186/s12958-024-01186-x

**Published:** 2024-01-22

**Authors:** Seung Hyun Park, Myung Chan Gye

**Affiliations:** Department of Life Science and Institute for Natural Sciences, Hanyang University, Seoul, 04763 Republic of Korea

**Keywords:** GSK3, Cyclophilin D, Motility regulation, Spermatozoa, Mouse

## Abstract

**Background:**

Cyclophilin D (CypD) negatively regulates ATP production by opening of the mitochondrial permeability transition pore. This study aimed to understand the role of CypD in sperm motility regulation.

**Methods:**

Changes in CypD during sperm capacitation and its interaction with glycogen synthase kinase 3α (GSK3α), a key kinase regulating sperm motility, were examined in mouse spermatozoa. The effects of CypD inhibitor cyclosporin A (CsA) and GSK3 inhibitor 6-bromo-indirubin-3'-oxime (BIO) on sperm motility, p-GSK3α(Ser21), mitochondrial permeability transition pore (mPTP), mitochondrial membrane potential (MMP), and ATP production were examined. The effect of proteasome inhibitor MG115 on the cellular levels of CypD was examined.

**Results:**

In cauda epididymal spermatozoa, GSK3α was found in both cytosolic and mitochondrial fractions whereas CypD was primarily found in the mitochondrial fraction together with ATP synthase F1 subunit alpha (ATP5A), a mitochondrial marker. GSK3α and CypD were co-localized in the sperm midpiece. Interaction between GSK3α and CypD was identified in co-immunoprecipitation. CsA, a CypD inhibitor, significantly increased sperm motility, tyrosine phosphorylation, mPTP closing, MMP, and ATP levels in spermatozoa, suggesting that CypD acts as a negative regulator of sperm function. Under capacitation condition, both GSK3α and CypD were decreased in spermatozoa but ATP5A was not. The GSK3 inhibitor BIO markedly increased p-GSK3α(Ser21) and decreased CypD but significantly increased mPTP closing, MMP, ATP production, and motility of spermatozoa. This suggests that inhibitory phosphorylation of GSK3α is coupled with degradation of CypD, potentiating the mitochondrial function. Degradation of CypD was attenuated by MG115, indicative of involvement of the ubiquitin proteasome system.

**Conclusions:**

During sperm capacitation, CypD act as a downstream target of GSK3α can be degraded via the ubiquitin proteasome system, stimulating mitochondrial function and sperm motility.

**Supplementary Information:**

The online version contains supplementary material available at 10.1186/s12958-024-01186-x.

## Introduction

Glycogen synthase kinase 3 (GSK3) is a serine/threonine kinase. Among the two GSK3 isoforms, GSK3α and GSK3β, GSK3α is a key regulator of sperm motility [3, 6, 18]. Inhibitory phosphorylation at Ser21 of GSK3α is crucial for sperm motility during epididymal sperm maturation and capacitation [7, 18, 27]. However, the mechanism regulating sperm motility downstream of GSK3α remains poorly understood. In cardiac myocytes and preodontoblasts, GSK3β up-regulates the opening of the mitochondrial permeability transition pore (mPTP), facilitating the formation of cyclophilin D (CypD) and adenine nucleotide translocator (ANT) complex. This results in mPTP opening [24, 25]. CypD as a sensitizer of the mPTP opening negatively regulates mitochondrial ATP production [1, 8]. This suggests that GSK3α may regulate sperm motility via participation in mPTP opening. Undoubtedly, ATP production is essential for activation of sperm motility and occurs primarily in mitochondria of the midpiece [2, 10, 16]. To elucidate the mechanism by which GSK3α regulates sperm motility, the interaction between CypD and GSK3α during sperm capacitation was investigated in mouse spermatozoa. During sperm capacitation, GSK3α interacts with CypD and enhances the degradation of CypD via the ubiquitin proteasome system, which stimulates sperm motility. To our knowledge, this is the first report that CypD as a downstream target of GSK3α regulates sperm motility.

## Materials and methods

### Ethics statement

Eight-week-old male ICR mice were obtained from DAEHAN Biolink (Eumsung, Korea). The mice were housed in the pathogen-free authorized facility at Hanyang University, where the temperature was 24 ± 2 ℃, the humidity was 50 ± 10%, and the dark/light cycle was 12 h. This study was approved by the Institutional Animal Care and Use Committee (IACUC) of Hanyang University (IACUC No. 2021-0134A).

### Sperm preparation and incubation

Cauda epididymides were isolated and decapsulated in phosphate-buffered saline (PBS) to eliminate blood. Spermatozoa were isolated from epididymides in Tyrode's basal medium (without Ca^2+^, bovine serum albumin [BSA], or bicarbonate) and centrifuged at 600 × g for 20 min in 35% Percoll. The pellets were washed in PBS and centrifuged at 800 × g for 10 min at 4℃. The sperm concentration was adjusted to 1 × 10^6^ cells/mL through the addition of Tyrode’s complete medium (TCM) containing with Ca^2+^, BSA, and bicarbonate. This medium condition spermatozoa to initiate capacitation. Sperm suspensions were incubated with GSK3 inhibitor 6-bromo-indirubin-3'-oxime (BIO), proteasome inhibitor Z-Leu-Leu-Norvalinal (MG115), and cyclosporin A (CsA) obtained from Sigma for 1 h in a CO_2_ incubator at 37 °C. After incubation, spermatozoa were subjected to motility analysis, protein isolation, and dry smear.

### Western blot analysis

Spermatozoa were homogenized in RIPA lysis buffer (WSE-7420; ATTO, Tokyo, Japan) containing 1% (v/v) protease and phosphatase inhibitor cocktail. After five rounds of sonication for 5 s at 4 °C, protein samples were obtained by centrifugation at 18,000× g for 20 min, and supernatants were subjected to western blot. Antibodies specific for rabbit anti GSK3α/β (#5676; Cell Signaling, Beverly, MA, USA), rabbit anti p-GSK3α/β (Ser21/9) (#9327; Cell Signaling), mouse anti cyclophilin D (ab110324; Abcam, Cambridge, UK), rabbit anti ATP synthase F1 subunit alpha (ATP5A) (ab176569; Abcam), rabbit anti β-tubulin (ab108342; Abcam), and rabbit anti GAPDH (sc-25778; SantaCruz Biotechnology, Dallas, TX, USA) were mixed in 5% skim milk in Tris-buffered saline containing 0.1% Tween 20 (TBST) and incubated with the membranes overnight at 4 °C. After being rinsed three times with TBST for 20 min, the membranes were incubated with peroxidase-conjugated anti-rabbit IgG (ab6721; Abcam) and anti-mouse IgG (ab6728; Abcam) mixed with skim milk in TBST for 1 h. After washing three times with TBST for 20 min, the signals were detected using ECL Reagent (RPN2232; Amersham Bioscience) and Fusion SL (Vilber Lourmat, Marne-la-Vallée, France). The relative band intensities were analyzed using ImageJ (Ver.1.51j8; National Institutes of Health) and are expressed in arbitrary units (AU).

### Immunocytochemistry

Dry spermatozoa smeared on poly-L-lysine-coated slides were fixed in acetone:methanol (1:1) solution at 4 °C for 10 min. Slides then were blocked in 5% donkey serum in 0.1% Triton X-100 PBS for 1 h and incubated with GSK3α/β, ATP5A, and cyclophilin D antibodies mixed in 1.5% donkey serum in a humidified chamber overnight at 4˚C. Rabbit IgG (ab172730; Abcam) and mouse IgG (sc-2023; Santa Cruz) were used as negative controls. After washing three times in PBS, slides were incubated with Alexa Fluor 488-conjugated anti-rabbit IgG (ab150061; Abcam) and Alexa Fluor 568-conjugated anti-mouse IgG (ab175472; Abcam) mixed in 1.5% donkey serum PBS for 1 h at room temperature. After washing three times in PBS, slides were mounted with DAPI (P36931; Invitrogen). Fluorescence images were captured by a microscope system with a cooled CCD (DP71; Olympus, Tokyo, Japan).

### Live mitochondria isolation

For isolation of live mitochondria in spermatozoa, the Mitochondria Isolation Kit (C3601; Beyotime Biotechnology, Shanghai, China) was used. Briefly, spermatozoa were suspended in cold mitochondrial isolation buffer containing 1 mM phenylmethanesulfonyl fluoride, incubated at 4℃ for 1 h, and punched 15 times with a cold glass homogenizer. Punched samples were centrifuged at 600 × g for 10 min and supernatants were collected. Acquired supernatants were centrifuged at 11,000 × g for 10 min at 4℃. The isolated mitochondria pellet was suspended in mitochondria digestion solution and subjected to western blot analysis together with cytosolic protein-containing supernatant.

### Co-immunoprecipitation assay

Spermatozoa were lysed in 0.1% Triton X-100 PBS and 1% protease and phosphatase inhibitor cocktails and subjected to co-immunoprecipitation (co-IP). Protein samples were precleared by protein A/G bead (sc-2003; Santa Cruz) at 4℃ for 2 h and obtained by centrifugation at 600 × g for 5 min. The supernatants were incubated with CypD antibodies and mouse IgG in a rolling machine at 4℃ overnight. The next day, protein A/G beads were added and incubated at 4℃ for 2 h. After washing five times in cold PBS by centrifugation at 600 × g for 5 min at 4℃, pellets were suspended in SDS-PAGE loading buffer and subjected to western blot analysis.

### Computer-assisted sperm analysis

Sperm motility was analyzed using video recording and computer-assisted sperm analysis (CASA). Briefly, 20-μL samples were released onto a MAKLER® counting chamber (Irvine Scientific, Santa Ana, CA, USA) at 37 °C. Videos were recorded by a Nikon Diaphot microscope system with a CoolSnap EZ CCD camera (Photometrics, Tucson, AZ, USA) using iSPERM software (CNC Biotech, Suwon, Korea). Sperm motility was measured at least 200 spermatozoa.

### Mitochondrial membrane potential assay

Mitochondrial membrane potential (MMP) of spermatozoa was measured using a tetramethylrhodamine, ethyl ester (TMRE) assay kit (ab113852; Abcam). Briefly, sperm were treated with TMRE and incubated for 30 min, and mesoxalonitrile 4-trifluoromethoxyphenylhydrazone was used as a negative control 10 min before TMRE treatment. Spermatozoa were washed in pre-warmed 0.2% BSA PBS and obtained by centrifugation at 500 g for 5 min. After centrifugation, the supernatant was discarded, and the pellet was suspended in PBS. TMRE fluorescence in spermatozoa was measured with a microplate reader (Varioskan; Thermo Scientific, Waltham, MA, USA) with an excitation wavelength of 549 nm and an emission wavelength of 575 nm. For TMRE fluorescence examination in spermatozoa, images were captured with a fluorescence microscope system with cooled CCD (DP71; Olympus).

### Measurement of mPTP opening

To measure mPTP opening in spermatozoa cobalt quenching of calcein fluorescence method was conducted [26]. Briefly, spermatozoa were incubated in media containing 1 μM calcein AM (Sigma) and 1 mM CoCl_2_ (Sigma) for 30 min at 36℃ and washed in PBS by centrifugation at 500 g for 5 min. The spermatozoa were resuspended in PBS, and calcein fluorescence was measured using a microplate reader (Varioskan; Thermo Scientific) with an excitation wavelength of 496 nm and an emission wavelength of 516 nm. For calcein fluorescence examination in spermatozoa, images were captured with a fluorescence microscope system with cooled CCD (DP71; Olympus).

### ATP assay

Sperm ATP level was analyzed using the CellTiter-Glo ® kit (G7570; Promega, Madison, WI, USA), which uses the enzymatic activity of the luciferase. Briefly, an equal volume of reagent was added to medium containing spermatozoa. The mixtures were shaking-incubated for 2 min to induce cell lysis and incubated at room temperature for 10 min to stabilize the luminescent signal. The luminescence of oxyluciferin was measured with a microplate reader (Varioskan) under an emission wavelength of 570 nm. Luminescence values are presented as relative light units (RLU).

### Statistical analysis

Student’s *t*-test and one-way analysis of variance (ANOVA) followed by Tukey’s test were used to evaluate statistical significance with SPSS (version 17.0; SPSS Inc, Chicago, IL, USA). Significant difference was determined at *p* < 0.05.

## Results

### Expression of CypD and GSK3 in spermatozoa

**Fig. 1:**
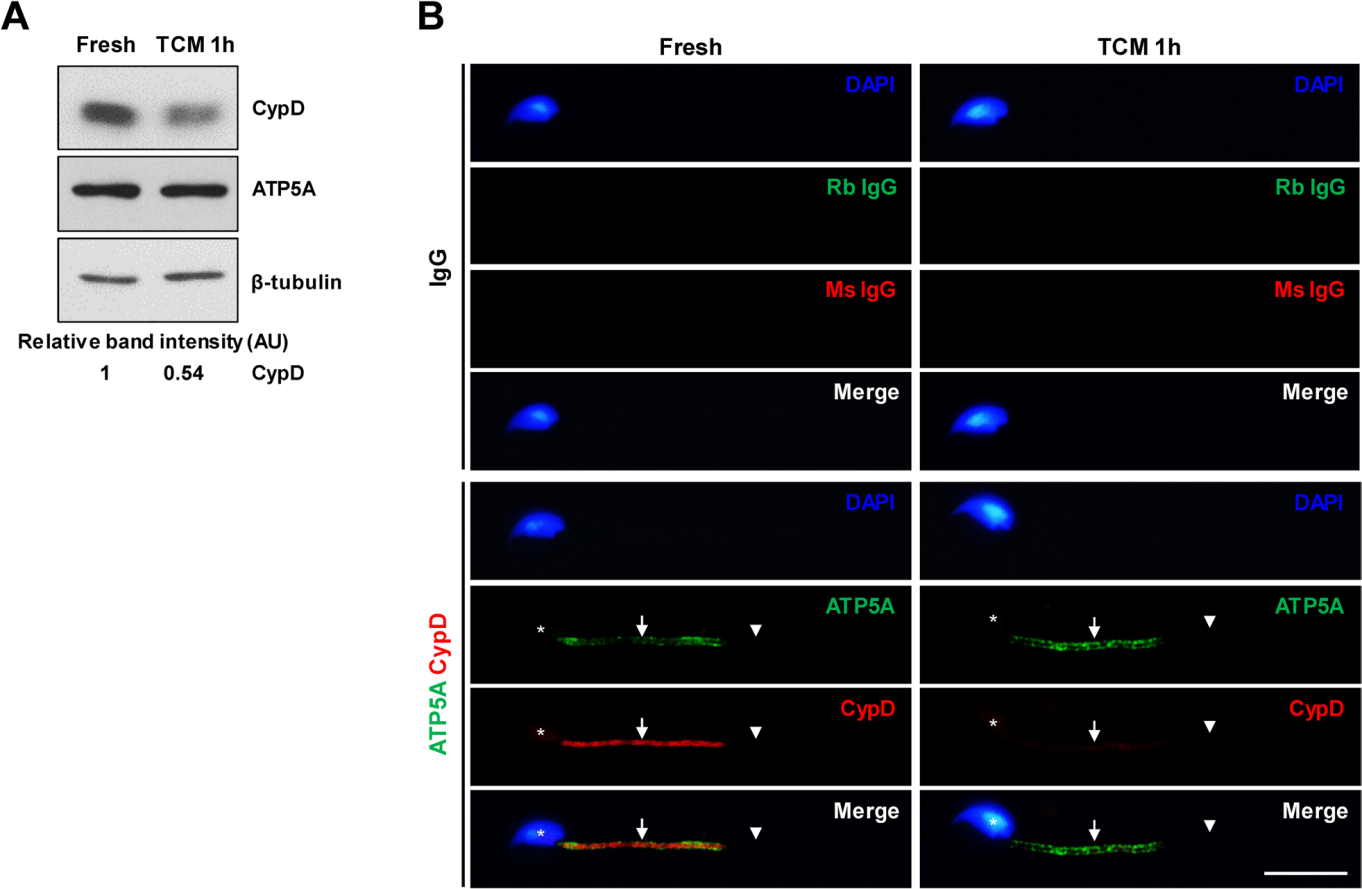
Change in CypD in cauda epididymal spermatozoa after capacitation. **A** Representative western blot images of changes in CypD and ATP5A proteins in spermatozoa after incubation in TCM for 1 h. CypD but not ATP5A levels were markedly decreased under capacitation condition. At least three independent experiments were subjected to western blot analysis. **B** Representative images of immunolocalization of CypD and ATP5A in cauda epididymal spermatozoa. Immunoreactivity of CypD was found in the midpiece and was decreased after incubation in TCM for 1 h. Rabbit and mouse IgG were used as a negative control of ATP5A and CypD antibodies, respectively. Sperm head, midpiece, and principal piece of the tail are annotated by asterisk, arrows, and arrowheads, respectively. Nuclei were stained blue by DAPI. At least three independent experiments were subjected to immunocytochemistry. AU, arbitrary units. Bar, 10 μm

**Fig. 2:**
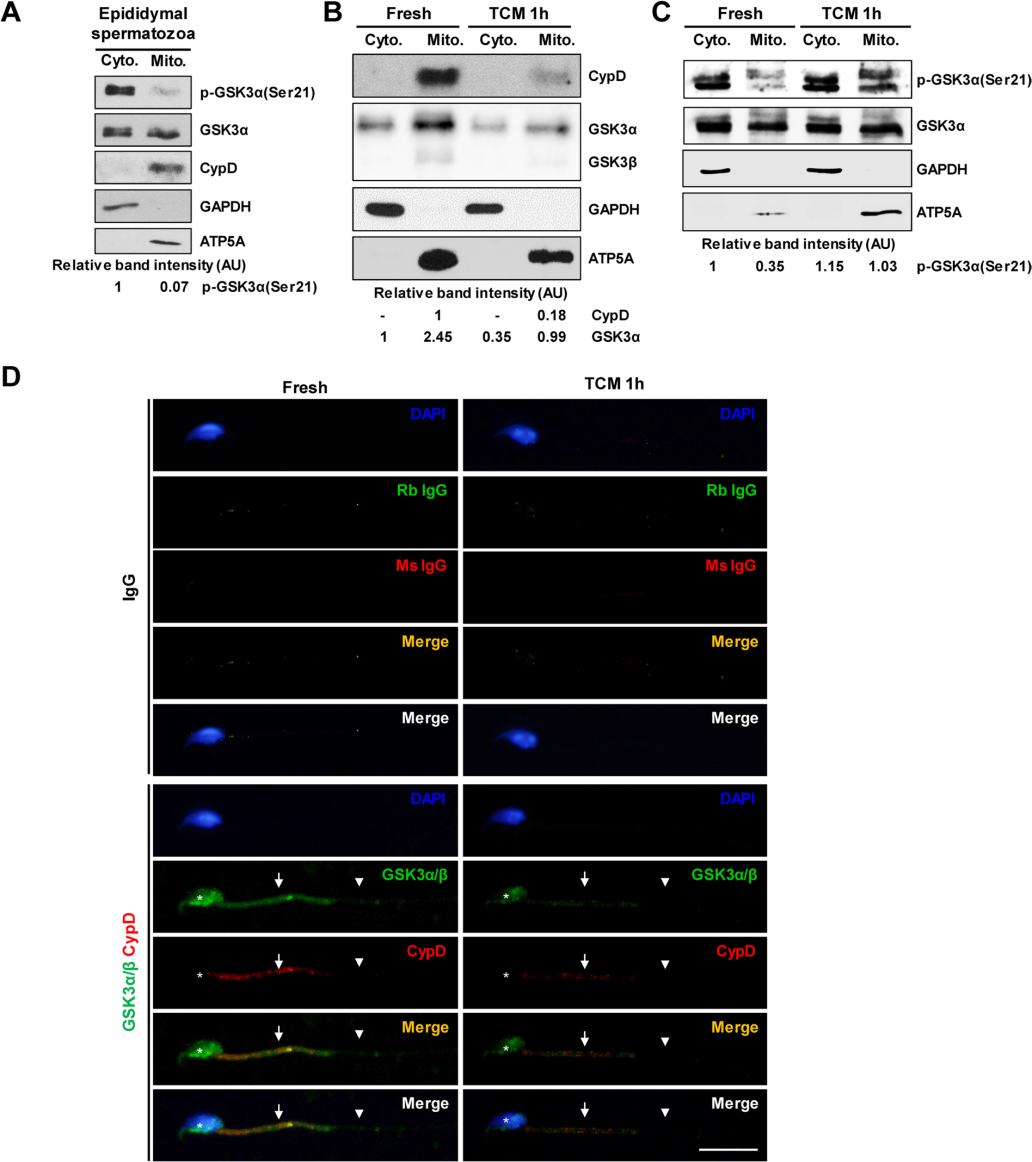
Expression of GSK3α and CypD in sperm mitochondria. **A** Representative western blot images of GSK3α and CypD expression in mitochondrial and cytosolic fractions after incubation in TCM for 1 h. GSK3α protein was found in both cytosol (Cyto) and mitochondrial (Mito) fractions, and p-GSK3α(Ser21) was higher in the cytosol fraction than in the mitochondrial fraction. In addition, CypD was only found in the mitochondria fraction. At least three independent experiments were subjected to western blot analysis. **B** Representative western blot images of changes in cellular amount of CypD and GSK3α in sperm cytosol and mitochondria during capacitation. CypD and GSK3α were markedly decreased after incubation in TCM for 1 h. ATP5A and GAPDH are mitochondria and cytosol markers, respectively. At least three independent experiments were subjected to western blot analysis. **C** Representative western blot images of change in p-GSK3α(Ser21) level in cytosol and mitochondria during capacitation. The p-GSK3α(Ser21) levels in cytosol and mitochondria were markedly increased during capacitation. At least three independent experiments were subjected to western blot analysis. **D** Representative images of immunolocalization of GSK3α/β and CypD in cauda epididymal spermatozoa. Immunoreactivity of GSK3α/β was found in the head, midpiece, and principal piece of the tail. Immunoreactivity of CypD was co-localized in the midpiece with GSK3. Immunoreactivity of both GSK3α/β and CypD was decreased after incubation in TCM for 1 h. Rabbit and mouse IgG were used as a negative control of GSK3α/β and CypD antibodies, respectively. Sperm head, midpiece, and principal piece of the tail are annotated by asterisk, arrows, and arrowheads, respectively. Nuclei were stained blue by DAPI. At least three independent experiments were subjected to immunocytochemistry. AU, arbitrary units. Bar, 10 μm

CypD but not mitochondrial marker ATP5A was markedly decreased in spermatozoa under capacitation condition. In double-labeled immunocytochemistry, strong immunoreactivity of CypD and ATP5A was found in the midpiece. The immunoreactivity of CypD but not ATP5A was markedly decreased under capacitation condition (Fig. 1). GSK3α was found in equal amounts in cytosolic and mitochondrial fractions, but p-GSK3α(Ser21) was higher in the cytosolic fraction than in the mitochondrial fraction. CypD and ATP5A protein were only detected in the mitochondrial fraction, while GAPDH was only found in the cytosolic fraction (Fig. 2A). In sperm lysates, CypD and GSK3α levels were markedly decreased after capacitation (Fig. 2B). In sperm lysates, p-GSK3α(Ser21) was markedly increased in both cytosolic and mitochondrial fraction, with a more pronounced increase observed in the mitochondrial fraction (Fig. 2C). In cauda epididymal spermatozoa, immunoreactivity of GSK3 was found in the sperm head, midpiece, and principal piece of the tail and was co-localized with immunoreactivity of CypD in the midpiece. The immunoreactivity of GSK3 was strong in the post-acrosomal region of the head, moderate in the midpiece, and weak to marginal in the acrosomal region of the head and principal piece of the tail. Immunoreactivity of GSK3 was markedly decreased in the sperm head, midpiece, and principal piece after capacitation. In the midpiece, immunoreactivity of CypD was markedly decreased after capacitation (Fig. 2D).

### Co-immunoprecipitation of CypD with GSK3α

**Fig. 3:**
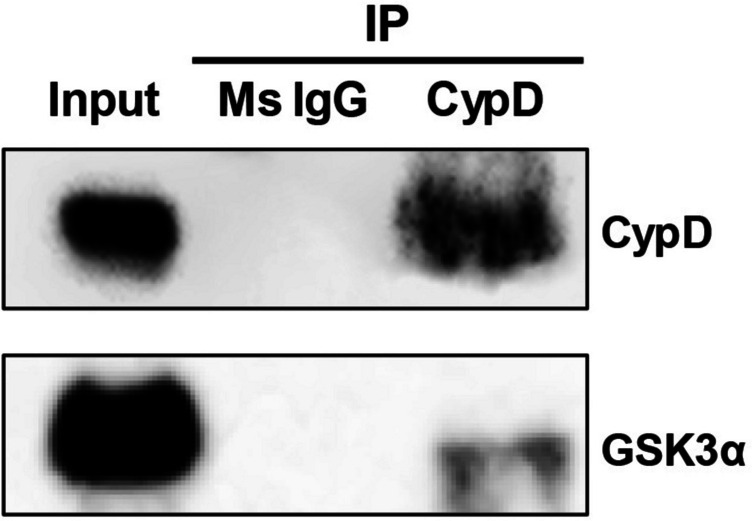
Interaction of GSK3α and CypD proteins in mouse spermatozoa. In co-immunoprecipitation of CypD in cauda epididymal spermatozoa, GSK3α interacts with CypD in mitochondria. In co-IP using normal IgG instead of CypD antibody, no signal was detected. Total sperm lysates were loaded in the input lane before co-IP to confirm the expression of CypD and GSK3α in spermatozoa

In co-IP, CypD protein was co-immunoprecipitated with GSK3α protein in spermatozoa. In co-IP using normal IgG instead of CypD antibody, no signal was detected (Fig. 3).

### Change in sperm motility, phosphotyrosine proteins, mPTP opening, MMP, p-GSK3α(Ser21), and ATP production by cyclosporin A, a CypD inhibitor

**Fig. 4:**
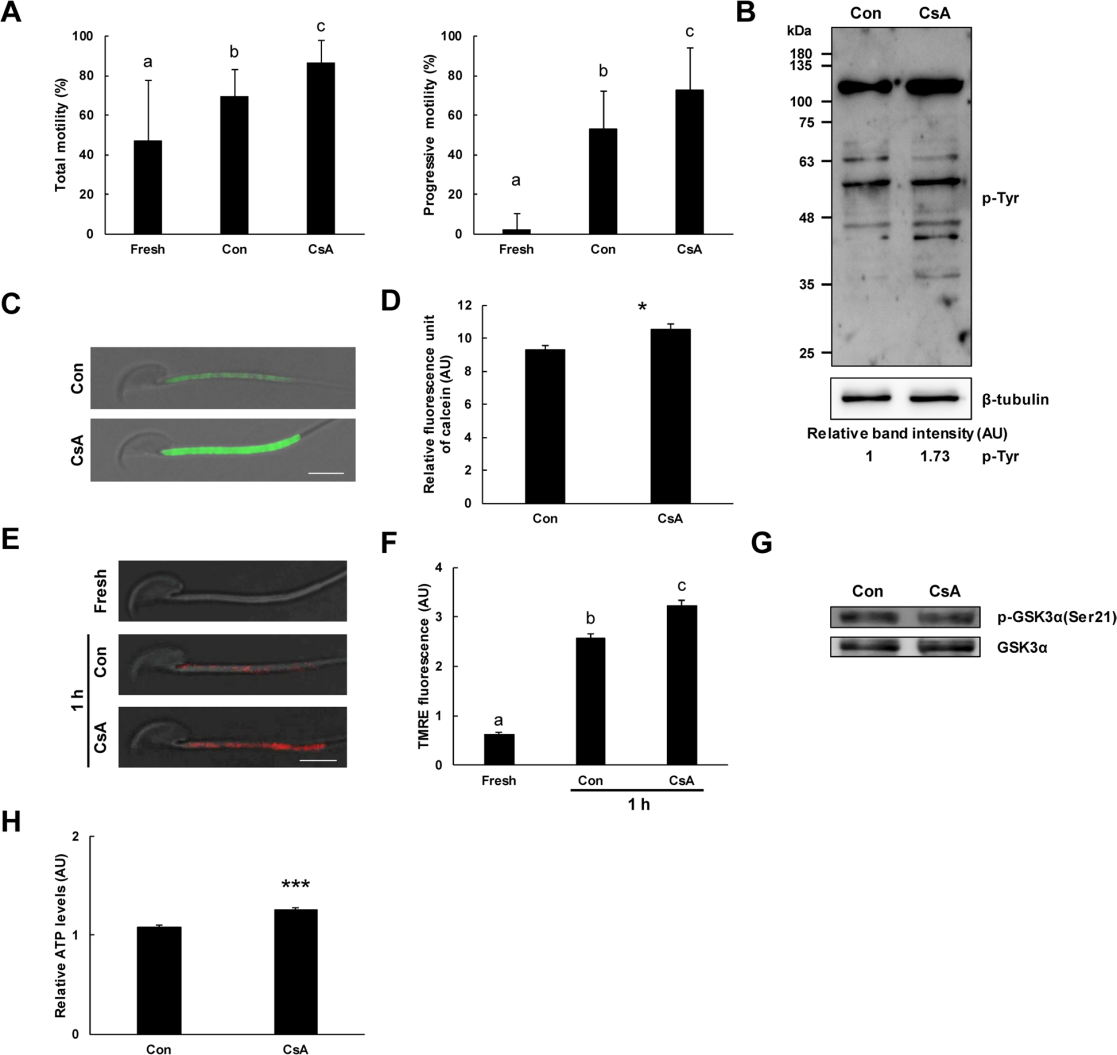
Effects of cyclosporin A, a CypD inhibitor on sperm motility, phosphotyrosine proteins, mPTP opening, p-GSK3α(Ser21), MMP, and ATP production. **A** Effects of cyclosporin A (CsA) on sperm motility. Total and progressive motility of spermatozoa were significantly increased under capacitation condition. CsA significantly increased total and progressive motility of spermatozoa (*n* = 4). **B** Representative western blot images of the effects of CsA on phosphotyrosine (p-Tyr) proteins of spermatozoa. CsA markedly increased p-Tyr proteins of spermatozoa. At least three independent experiments were subjected to western blot analysis. **C** Representative images of calcein fluorescence in CsA-treated spermatozoa. CsA markedly increased calcein fluorescence intensity in the sperm midpiece. Bar, 5 μm. **D** Effect of CsA on mPTP opening. CsA significantly increased calcein fluorescence, indicative of decreased mPTP opening in spermatozoa under capacitation condition (*n* = 4). **E** Representative images of TMRE fluorescence in CsA-treated spermatozoa. TMRE fluorescence intensity was markedly increased in the sperm midpiece under capacitation and was further potentiated by CsA. Bar, 5 μm. **F** Effects of CsA on sperm mitochondrial membrane potential (MMP). MMP of spermatozoa was significantly increased under capacitation condition. CsA significantly increased the MMP of spermatozoa (*n* = 4). **G** Effect of CsA on p-GSK3α(Ser21). No change was observed in p-GSK3α(Ser21) level by CsA treatment in spermatozoa under capacitation condition. At least three independent experiments were subjected to western blot analysis. **H** Effect of CsA on ATP production in spermatozoa. CsA significantly increased ATP production in spermatozoa (*n* = 4). Data are presented as mean ± SD. Significant differences on one-way ANOVA (*p* < 0.05) are indicated by different letters. *p < 0.05 and ***p < 0.001 indicates significant difference from control by Student’s *t*-test. AU, arbitrary units

In CASA, the cyclosporin A (CsA) (10 μM) CypD inhibitor significantly increased total and progressive motility of spermatozoa (Fig. 4A). In western blot, CsA markedly increased phosphotyrosine (p-Tyr) proteins, a capacitation marker (Fig. 4B). CsA significantly increased calcein fluorescence in sperm midpiece, indicative of decreased mPTP opening in spermatozoa (Fig. 4C and D). TMRE fluorescence intensity in sperm midpiece was significantly increased during sperm capacitation and was potentiated by CsA (Fig. 4E and F). No change was found in p-GSK3α(Ser21) level after CsA treatment (Fig. 4G). CsA significantly increased ATP production in spermatozoa (Fig. 4H).

### Change in p-GSK3α(Ser21), motility, CypD, mPTP opening, MMP, and ATP production by BIO, a GSK3 inhibitor

**Fig. 5:**
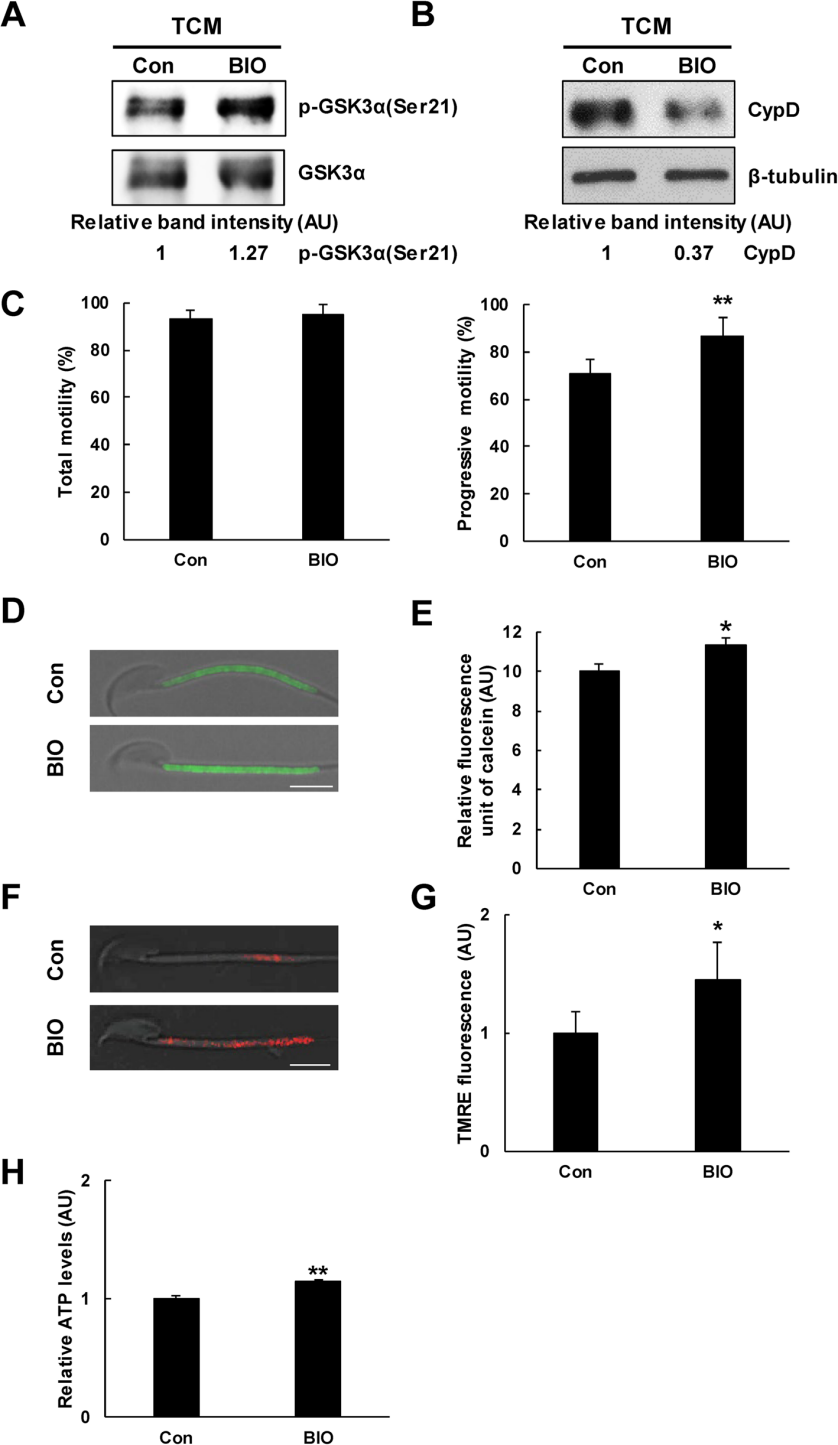
Effects of BIO, a GSK3 inhibitor on p-GSK3α(Ser21), CypD, mPTP opening, MMP, and ATP production. **A** Representative western blot images of the effect of GSK3 inhibitor BIO on inhibitory phosphorylation of GSK3α. GSK3 inhibitor BIO increased p-GSK3α(Ser21) in spermatozoa. At least three independent experiments were subjected to western blot analysis. **B** Representative western blot images of the effect of GSK3 inhibitor BIO on amount of CypD. GSK3 inhibitor BIO decreased the protein amount of CypD in spermatozoa. At least three independent experiments were subjected to western blot analysis. (**C**) Effect of GSK3 inhibitor BIO on sperm motility. BIO significantly increased progressive motility but not total motility (*n* = 4). **D** Representative images of calcein fluorescence in BIO-treated spermatozoa. BIO markedly increased calcein fluorescence intensity in the sperm midpiece. Bar, 5 μm. **E** Effect of BIO on mPTP opening. BIO significantly increased calcein fluorescence, indicative of decreased mPTP opening in spermatozoa under capacitation condition (*n* = 4). **F** Representative images of TMRE fluorescence in BIO-treated spermatozoa. BIO markedly increased TMRE fluorescence intensity in the sperm midpiece. Bar, 5 μm. **G** Effect of GSK3 inhibitor BIO on sperm mitochondrial membrane potential (MMP). BIO significantly increased sperm MMP (*n* = 4). **H** Effect of GSK3 inhibitor BIO on sperm ATP production. BIO significantly increased ATP production in spermatozoa (*n* = 4). Data are presented by mean ± SD. *p < 0.05 and **p < 0.05 indicate significant difference from control by Student’s *t*-test. AU, arbitrary units

In cauda epididymal spermatozoa, treatment with 500 nM BIO, a GSK3 inhibitor, markedly increased p-GSK3α(Ser21) (Fig. 5A). BIO markedly decreased cellular amount of CypD in spermatozoa (Fig. 5B). In CASA, BIO significantly increased progressive whereas no change was observed in total motility. BIO significantly increased calcein fluorescence in sperm midepiece, indicative of decreased mPTP opening in spermatozoa (Fig. 5D and E). BIO significantly increased TMRE fluorescence intensity in sperm midpiece (Fig. 5F and G). BIO significantly increased ATP levels in spermatozoa (Fig. 5H).

### Changes in CypD level and sperm motility after MG115 treatment

**Fig. 6:**
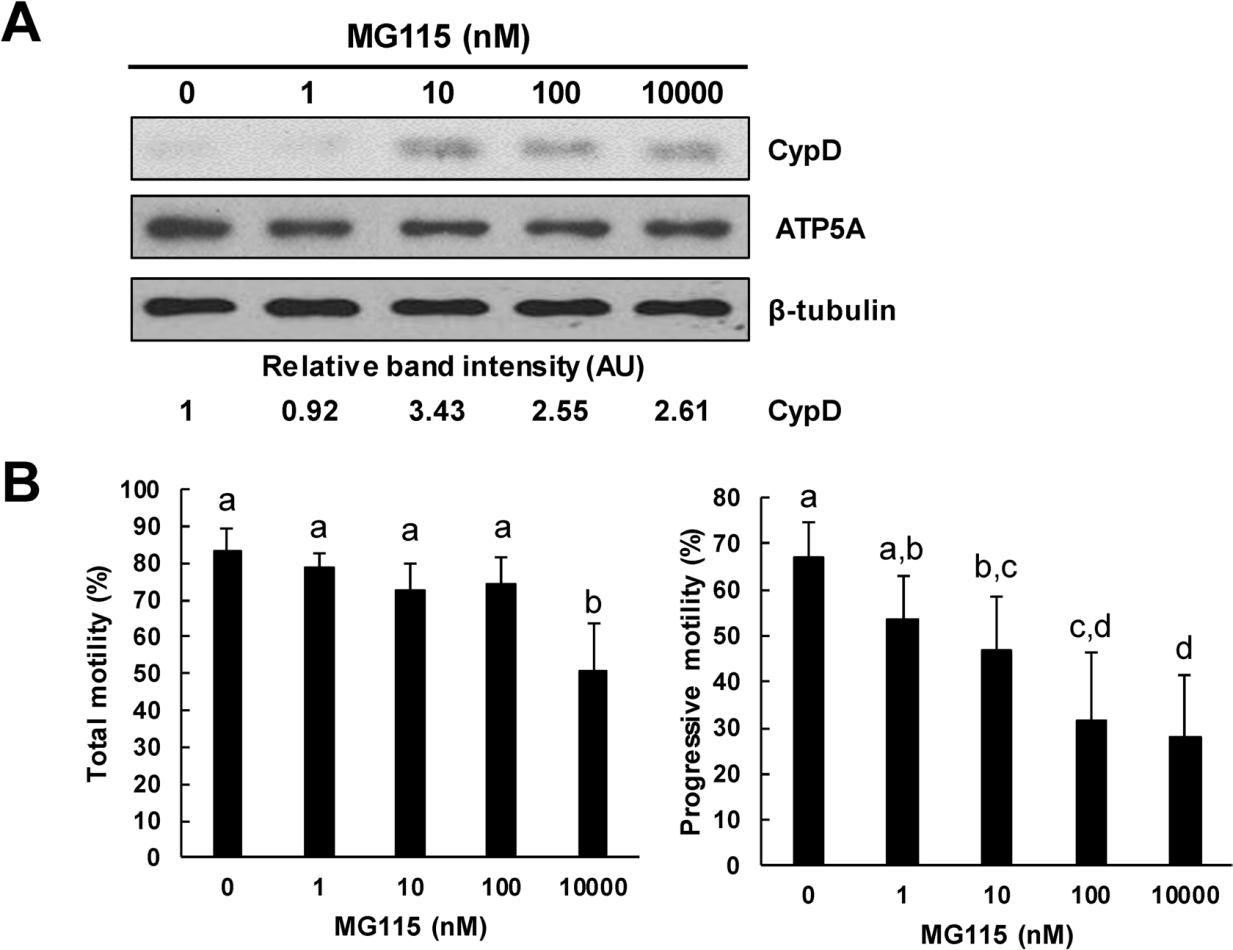
Effects of proteasome inhibitors on the cellular amount of CypD protein in mouse spermatozoa. **A** Representative western blot images of the effect of proteasome inhibitor MG115 on the amount of CypD protein. Proteasome inhibitor MG115 in TCM increased the amount of CypD protein in spermatozoa. No change is cellular amount of ATP5A, mitochondria marker was observed regardless of proteasome inhibitor treatment. At least three independent experiments were subjected to western blot analysis. **B** Effect of MG115 on sperm motility. MG115 significantly decreased total and progressive motility of spermatozoa (*n* = 4). Data are presented as mean ± SD. Significant differences are indicated by different letters using one-way ANOVA (*p* < 0.05). AU, arbitrary units

MG115 at 10 nM markedly increased CypD level in spermatozoa whereas no change was observed in ATP5A mitochondrial marker. In CASA, MG115 significantly decreased progressive and total motility of spermatozoa (Fig. 6).

## Discussion

### Changes in CypD and GSK3α in spermatozoa during capacitation

In mammalian spermatozoa, protein degradation by ubiquitination has been suggested [15, 28]. During sperm capacitation, CypD but not ATP5A was markedly decreased in the sperm midpiece. This suggests that CypD is vulnerable to proteasome degradation during capacitation in spermatozoa but ATP5A is not. This degradation of CypD may stimulate ATP production in spermatozoa, activating sperm motility during capacitation. In mammals, GSK3α is a well-known regulator of sperm motility [18, 27]. GSK3α was found in both cytosolic and mitochondrial fractions, whereas CypD was primarily found in the mitochondrial fraction. In fresh spermatozoa, CypD and GSK3α were co-localized in the midpiece, and the levels of CypD and GSK3α decreased during capacitation. In fresh spermatozoa, p-GSK3α(Ser21) level in the cytosolic fraction was higher than that of the mitochondrial fraction. Of note, p-GSK3α(Ser21) level increased in the mitochondrial fraction during capacitation. Given that decreased kinase activity of GSK3α through serine phosphorylation is crucial for sperm motility [7, 18, 27], this increase in mitochondrial p-GSK3α(Ser21) may support activation of sperm motility. In rat cardiomyoblast cells, translocation of GSK3β to mitochondria requires its kinase activity [23]. In fresh spermatozoa, the low level of inhibitory phosphorylation of mitochondrial GSK3α may reflect translocation of active GSK3α to mitochondria, in which active GSK3α may negatively regulate sperm motility. During capacitation, the amount of GSK3α was decreased together with an increase in p-GSK3α(Ser21) and a decrease in CypD in the mitochondrial fraction. This may result in decreased phosphorylation of GSK3α substrate proteins, including CypD, during capacitation. Together, these findings suggest that degradation of CypD and increase in p-GSK3α(Ser21) may stimulate sperm motility activation during capacitation in spermatozoa.

### Inhibition of CypD stimulates sperm motility

Mitochondrial activity is positively correlated with sperm motility [5, 22]. In mouse spermatozoa, the CypD inhibitor CsA increased sperm motility and p-Tyr proteins, a capacitation marker. This suggests that mitochondrial CypD directly regulates sperm motility and capacitation. MMP is a functional marker of ATP synthesis in mitochondria [29]. In the TMRE assay, CsA significantly increased MMP of spermatozoa. Additionally, CsA significantly increased ATP production and decreased mPTP opening in spermatozoa under capacitation condition independent of p-GSK3α(Ser21). This suggests that CypD negatively regulates mitochondrial ATP production in spermatozoa. In mammals, the control of motility and capacitation is essential due to the asynchrony between mating and fertilization in the female reproductive tract [11, 12, 17]. Together, CypD may inhibit mitochondrial ATP production, leading to the inhibition of sperm motility activation before capacitation.

### Role of GSK3α as a regulator of CypD in spermatozoa

In rodent cardiomyocytes and human embryonic kidney cells, GSK3β has been reported as a modulator of mPTP via phosphorylation of CypD (Ser191) [13]. The CypD-ANT complex has been identified as a key component of mPTP opening [24, 25]. In rat cardiomyocytes, p-GSK3β (Ser9) binds to ANT, decreasing the binding between CypD and ANT [24]. In the co-IP assay, GSK3α co-precipitated with CypD, indicative of direct interaction between GSK3α and CypD in spermatozoa. This suggests that GSK3α may participates in mPTP opening via phosphorylation of CypD, increasing the formation of ANT-CypD complex in mouse spermatozoa. The GSK3 inhibitor BIO markedly decreased CypD level in spermatozoa. Phosphorylation by GSK3 is important for protein degradation through the ubiquitin–proteasome system [9, 20, 21]. Given that CypD has GSK3-specific phosphorylation sites [13], serine phosphorylation of CypD by active GSK3α may inhibit proteasomal degradation of CypD in spermatozoa. Of note, BIO increased MMP and ATP levels and progressive motility and decreased mPTP opening of spermatozoa. This suggests that inhibitory phosphorylation of GSK3α potentiate mitochondrial activity via mPTP closing, leading to motility activation in spermatozoa. The proteasome inhibitor MG115 attenuated degradation of CypD and deceased motility in spermatozoa during capacitation. Although mitochondria do not contain proteasomes, they do have a protein export system, which enables removal of mitochondrial proteins [4, 14, 19]. This suggests that ubiquitin proteasome system-mediated CypD degradation occurs in the cytosol. Thus, downregulation of CypD phosphorylation, facilitated by inhibitory phosphorylation of GSK3, enhances the degradation of CypD, potentiating sperm motility during capacitation.

## Conclusions

**Fig. 7:**
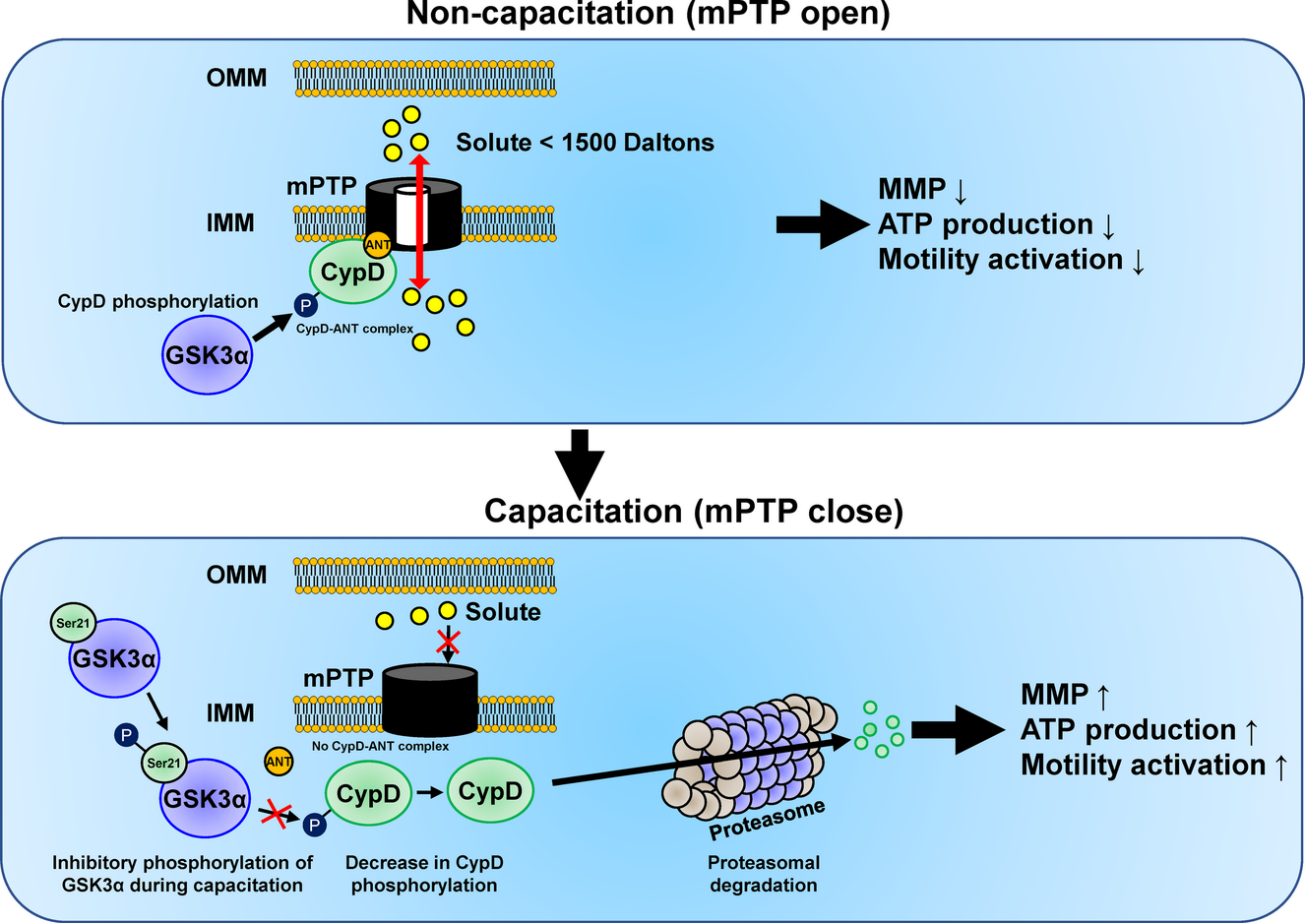
Roles of CypD in sperm motility regulation as a key regulator of GSK3α in spermatozoa. In fresh spermatozoa, GSK3α phosphorylates CypD, facilitating the formation of the CypD and ANT complex. This results in the opening of mPTP, decreasing MMP and ATP production and inhibits the activation of sperm motility. During sperm capacitation, inhibitory phosphorylation of GSK3α stimulates proteasomal degradation of CypD, inhibiting the mPTP opening, and which activates ATP production and sperm motility. ANT, adenine nucleotide translocator; mPTP, mitochondrial permeability transition pore; CypD, cyclophilin D; GSK3α, glycogen synthase kinase 3α; OMM, outer mitochondrial membrane; IMM, inner mitochondrial membrane; MMP, mitochondrial membrane potential; ATP, adenosine triphosphate

Inhibitory phosphorylation of GSK3α mediates proteasomal degradation of mitochondrial CypD, potentiating the mitochondrial ATP production through the mPTP closing. This results in activation of motility during sperm capacitation (Fig. 7).

### Supplementary Information

**Additional file 1.**

## Data Availability

No datasets were generated or analysed during the current study.
