# Supplementary material for: Inhibition of mitochondrial cyclophilin D, a downstream target of glycogen synthase kinase 3α, improves sperm motility
Source: Reprod Biol Endocrinol. 2024 Jan 22;22:15. doi: 10.1186/s12958-024-01186-x (PMC10802072; doi:10.1186/s12958-024-01186-x)

Full uncropped blots of Figure 1A

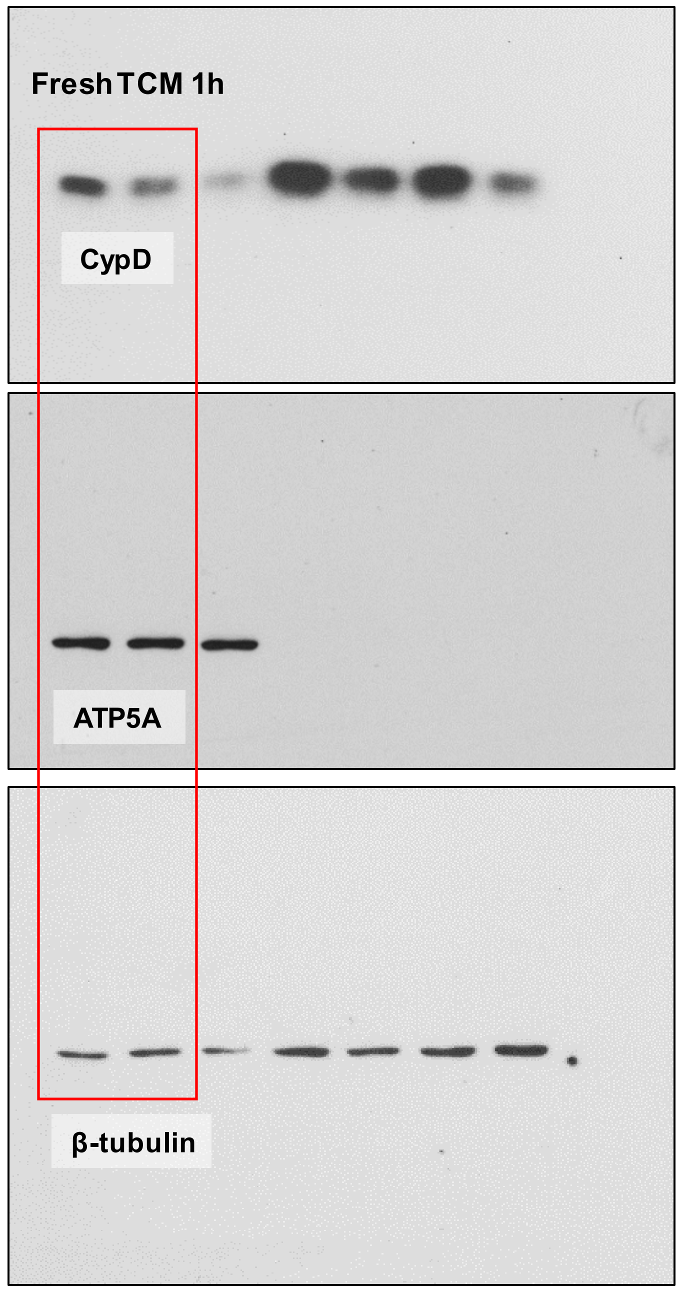

Full uncropped blots of Figure 2A

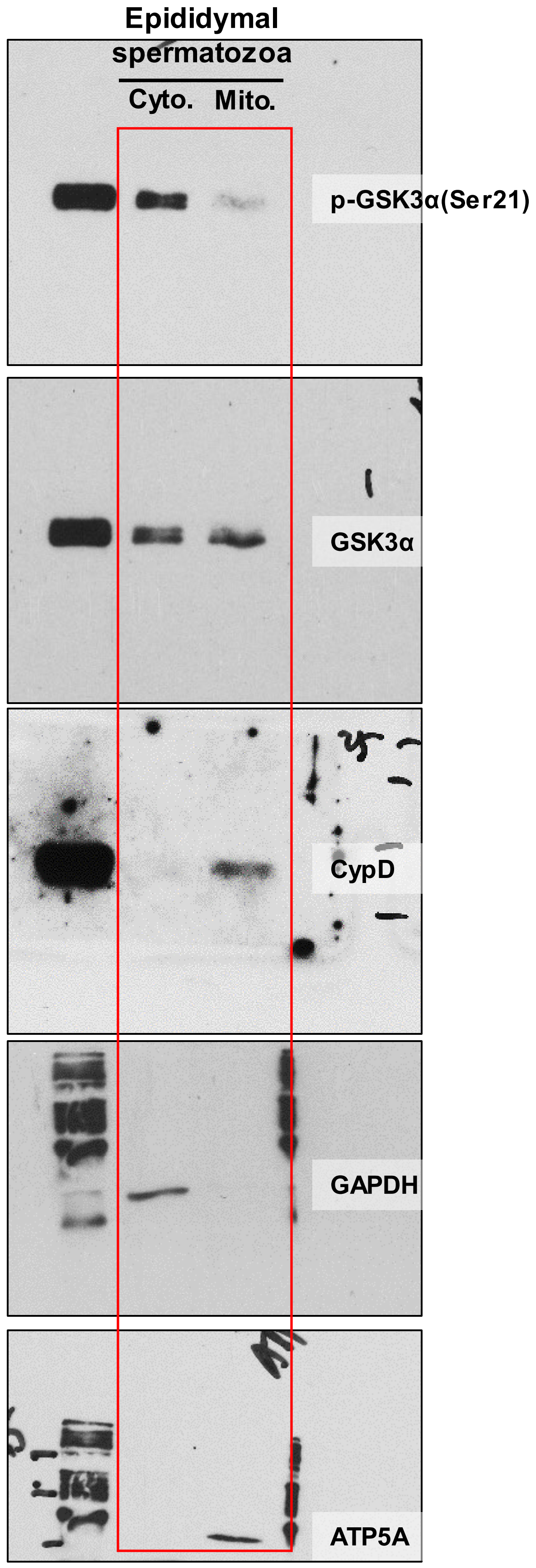

Full uncropped blots of Figure 2B

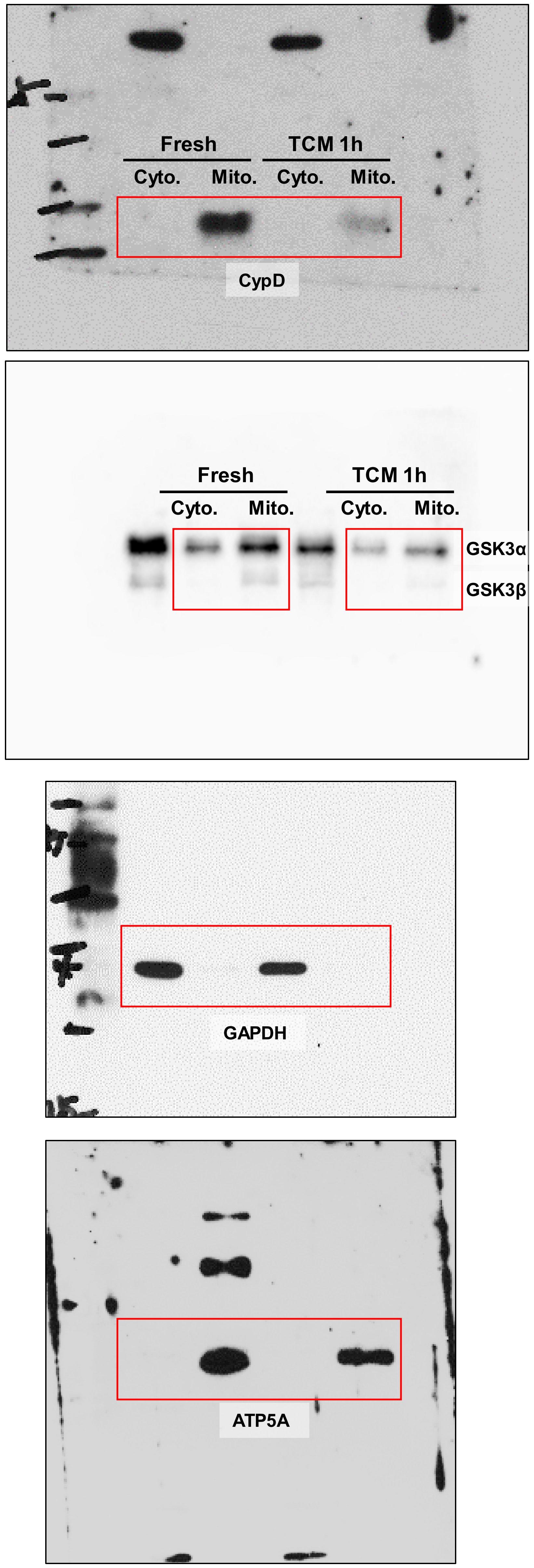

Full uncropped blots of Figure 2C

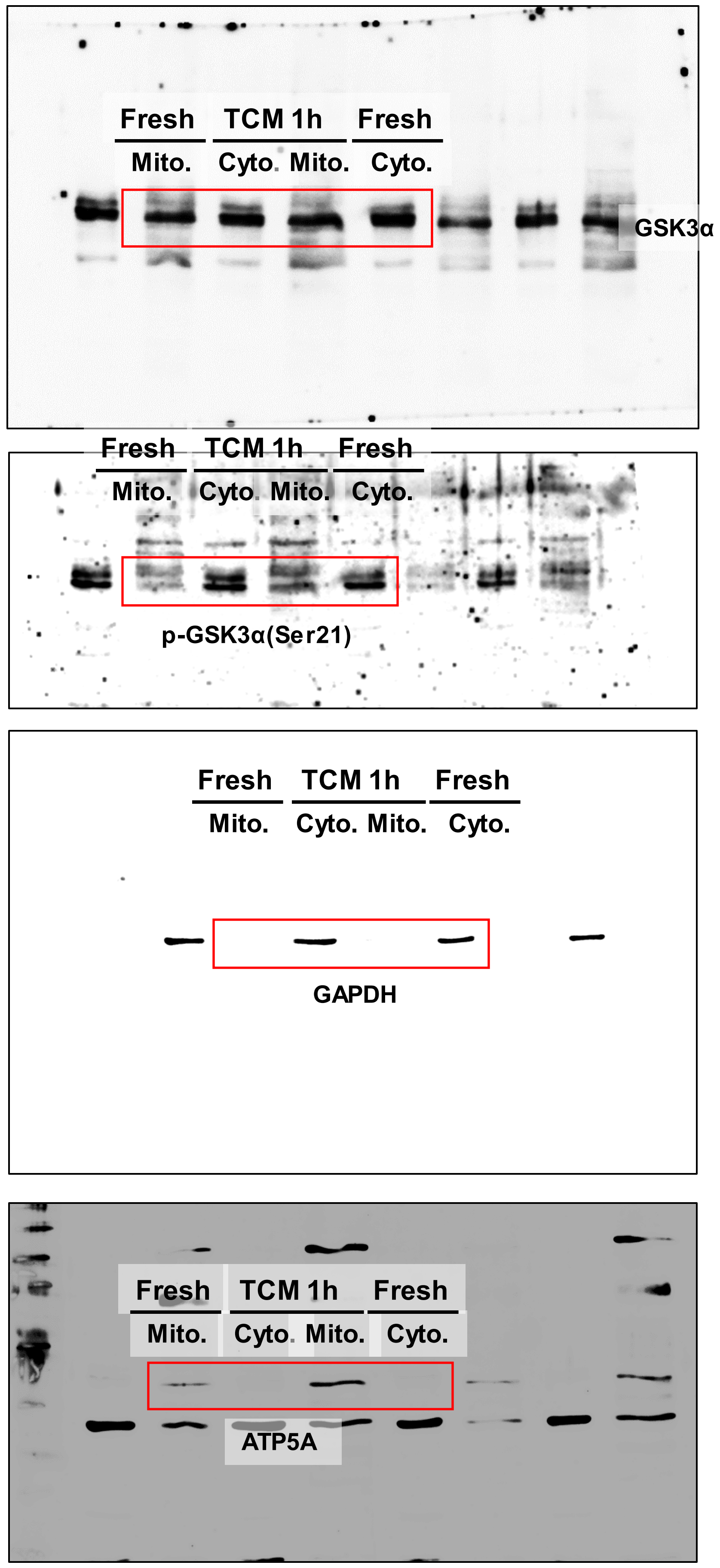

Full uncropped blots of Figure 3

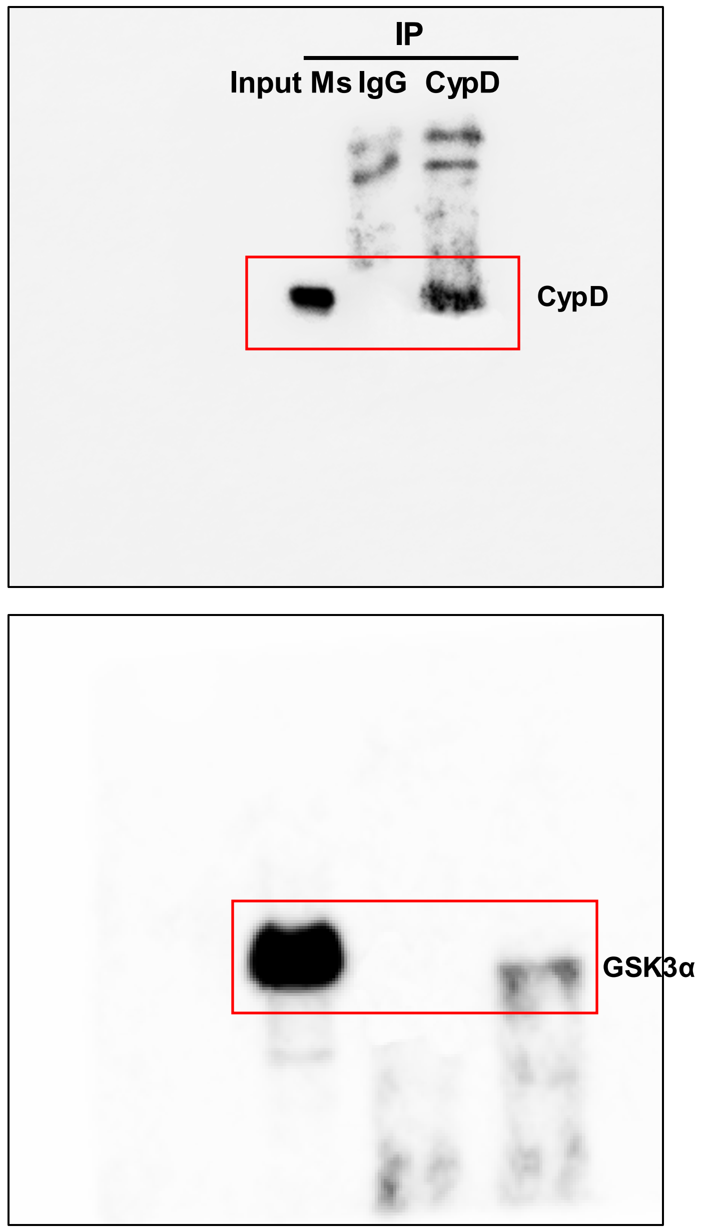

Full uncropped blots of Figure 4B

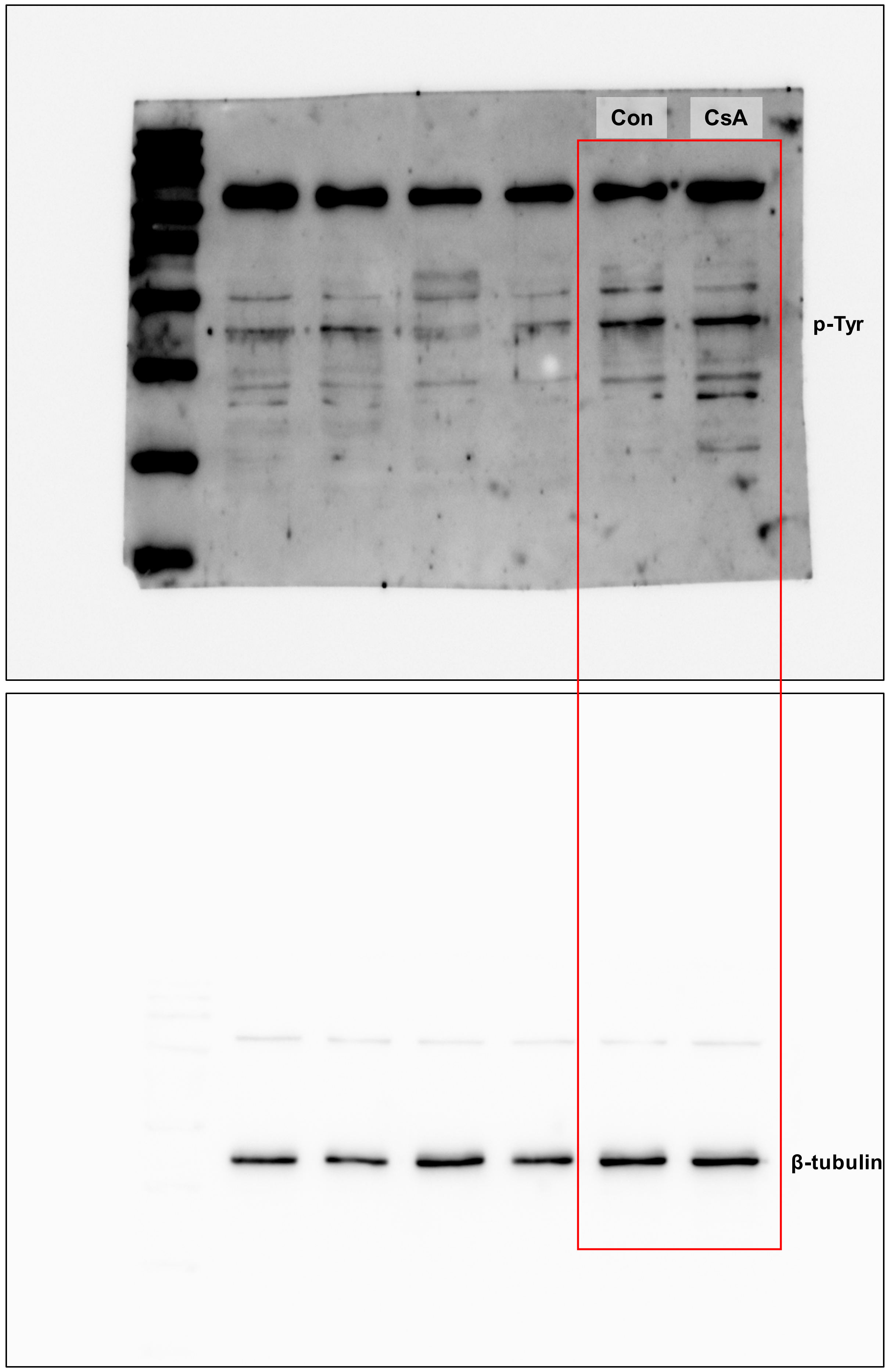

Full uncropped blots of Figure 4G

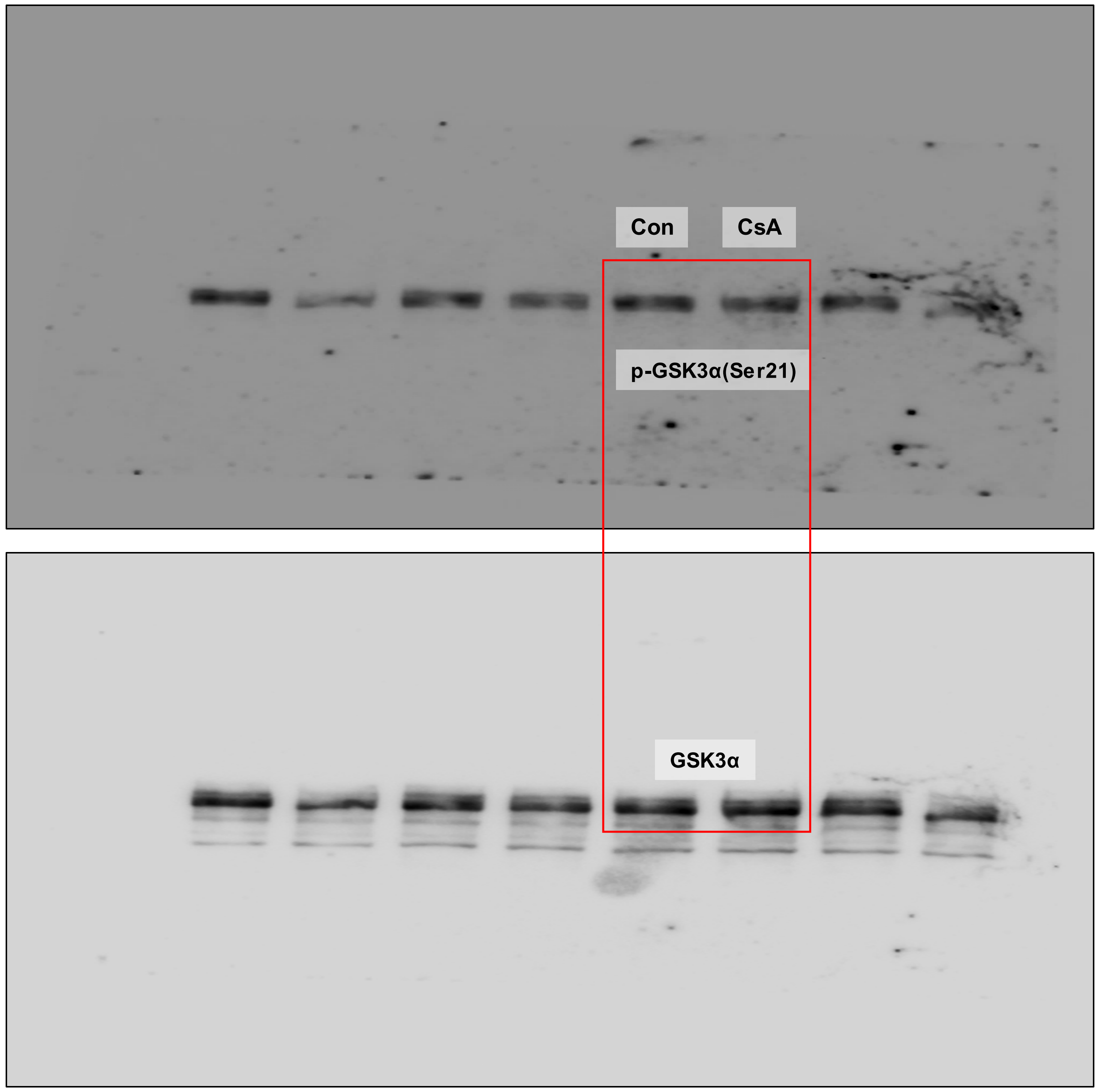

Full uncropped blots of Figure 5A

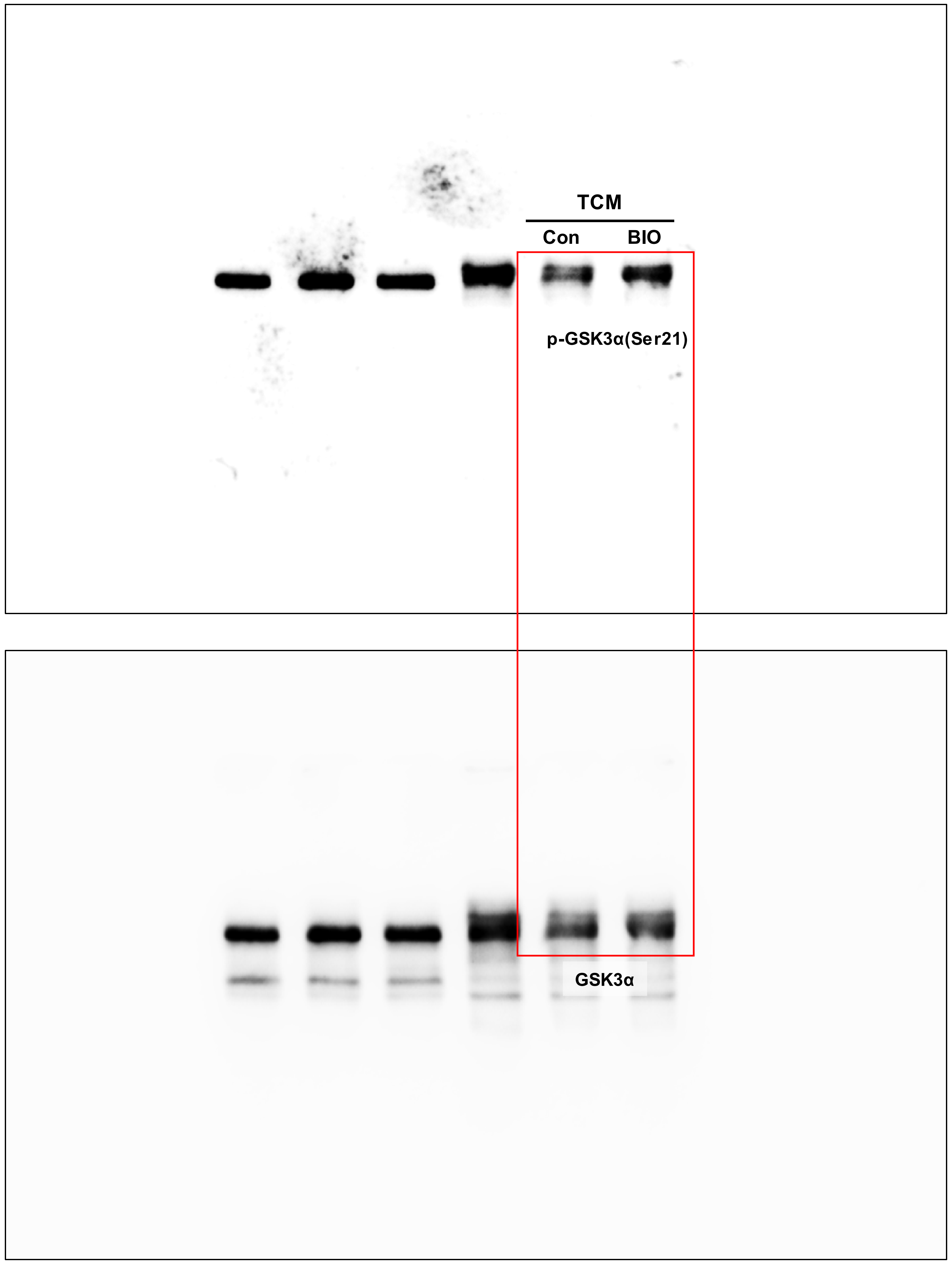

Full uncropped blots of Figure 5B

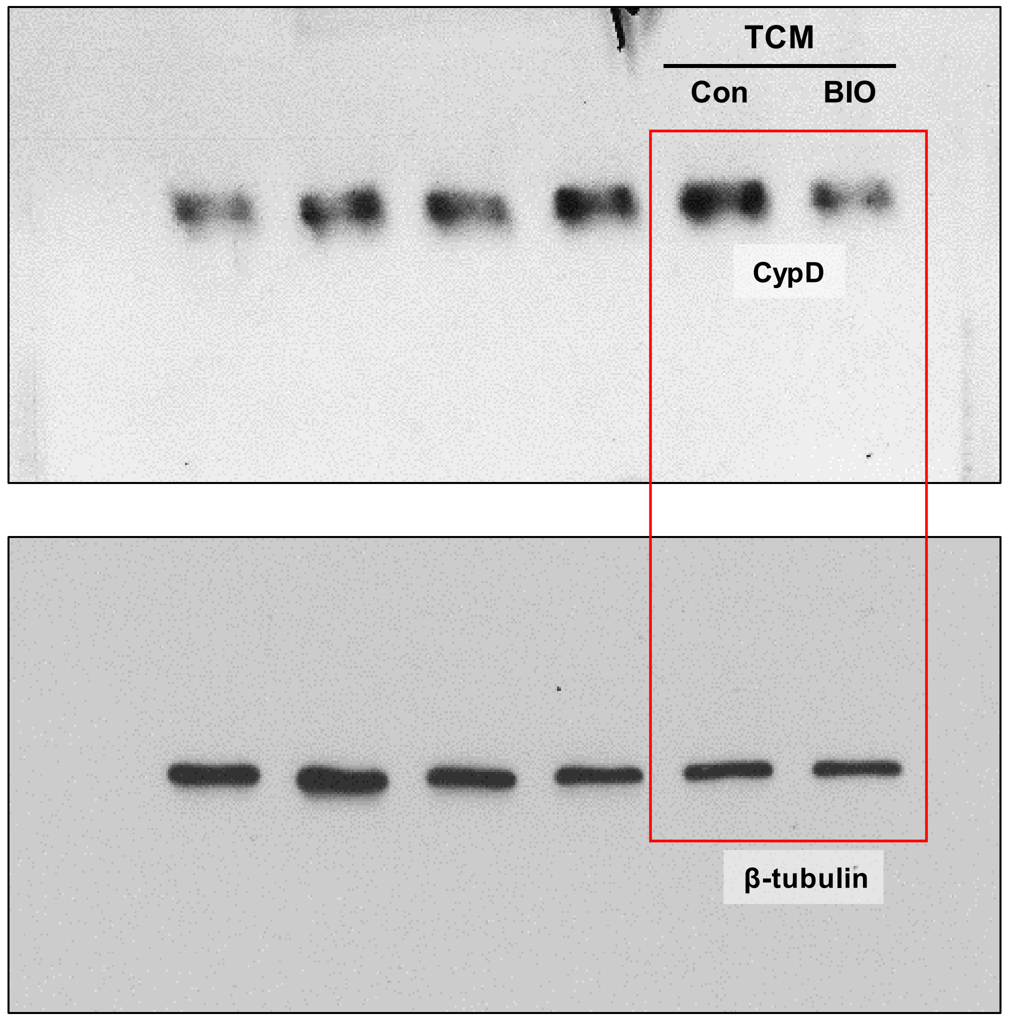

Full uncropped blots of Figure 6A

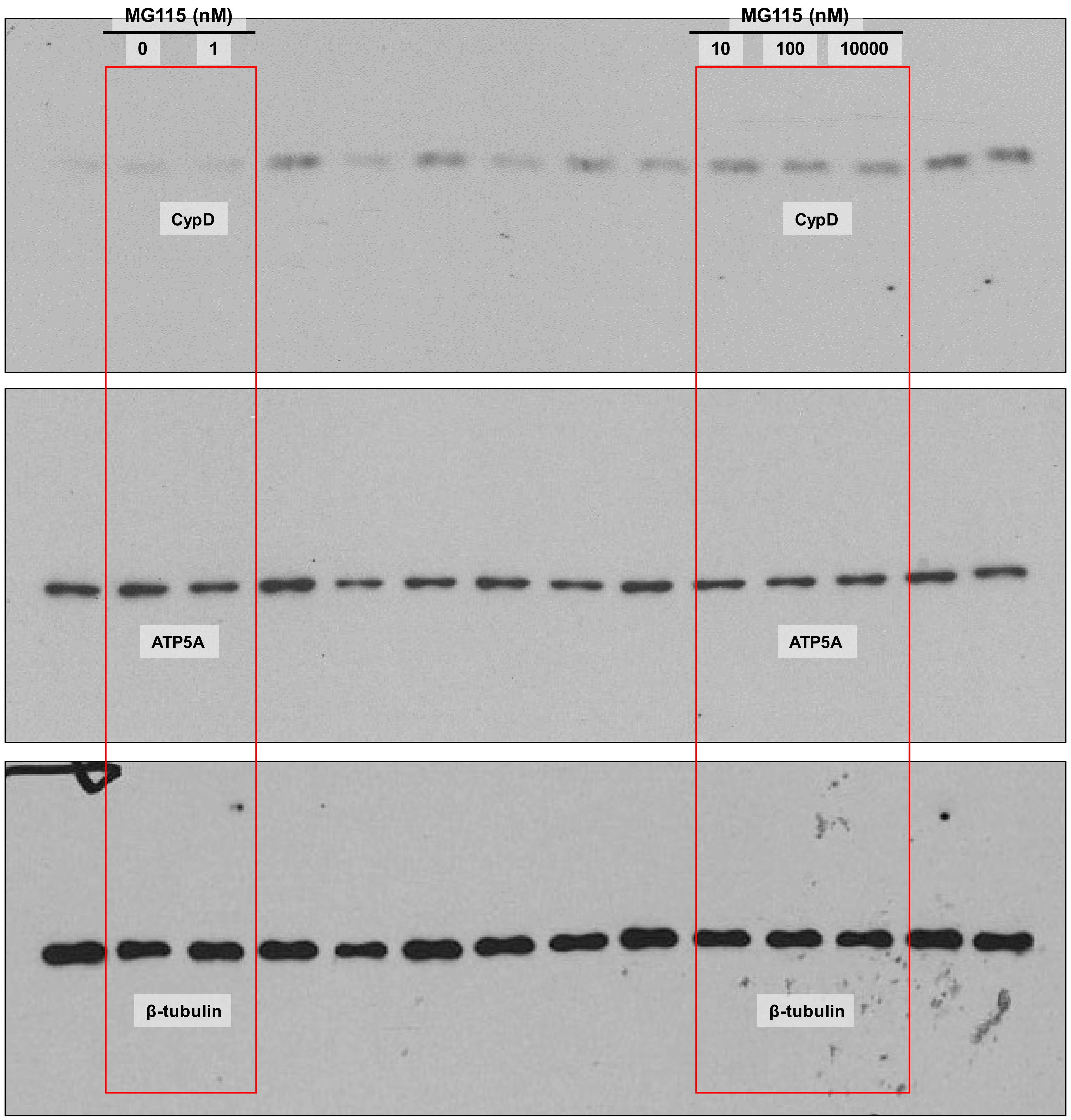

Supplement: Supplementary file 1 — Additional file 1. [file 12958_2024_1186_MOESM1_ESM.pdf]
